# Clinical and Bacterial Characteristics of *Klebsiella pneumoniae* Affecting 30-Day Mortality in Patients With Bloodstream Infection

**DOI:** 10.3389/fcimb.2021.688989

**Published:** 2021-09-16

**Authors:** Xingbing Wu, Qingyi Shi, Shimo Shen, Chen Huang, Hongcheng Wu

**Affiliations:** ^1^Department of Infectious Diseases, Ningbo Medical Center Lihuili Hospital, Ningbo, China; ^2^Department of Rheumatology, Ningbo Medical Center Lihuili Hospital, Ningbo, China; ^3^Department of Respiratory Medicine, Ningbo Medical Center Lihuili Hospital, Ningbo, China

**Keywords:** *Klebsiella pneumoniae*, bloodstream infection (BSI), mortality, hypervirulent, carbapenem-resistant, risk factors

## Abstract

**Background:**

There is a paucity of studies using clinical characteristics and whole-genome sequencing together to fully identify the risk factors of patients with *Klebsiella pneumoniae* (KP) bloodstream infection (BSI).

**Methods:**

We retrospectively analyzed the clinical and microbiological characteristics of patients with KP BSI. Isolates were processed using Illumina NGS, and relevant bioinformatics analysis was conducted (multi-locus sequence typing, serotype, phylogenetic reconstruction, detection of antibiotic resistance, and virulence genes). A logistic regression model was used to evaluate the risk factors of hosts and causative KP isolates associated with 30-day mortality in patients infected with KP BSI.

**Results:**

Of the 79 eligible patients, the 30-day mortality rate of patients with KP BSI was 30.4%. Multivariate analysis showed that host-associated factors (increased APACHE II score and septic shock) were strongly associated with increased 30-day mortality. For the pathogenic factors, carriage of *iutA* (OR, 1.46; 95% CI, 1.11–1.81, *p* = 0.002) or *Kvar_1549* (OR, 1.31; 95% CI, 1.02–1.69, *p* = 0.043) was an independent risk factor, especially when accompanied by a multidrug-resistant phenotype. In addition, ST11-K64 hypervirulent carbapenem-resistant KP co-harbored acquired *bla_KPC-2_* together with *iutA* (76.5%, 13/17) and *Kvar_1549* (100%, 17/17) genes. Comparative genomic analysis showed that they were clustered together based on a phylogenetic tree, and more virulence genes were observed in the group of ST11-K64 strains compared with ST11-non-K64. The patients infected with ST11-K64 strains were associated with relatively high mortality (47.2%, 7/17).

**Conclusion:**

The carriage of *iutA* and *Kvar_1549* was seen to be an independent mortality risk factor in patients with KP BSI. The identification of hypervirulent and carbapenem-resistant KP strains associated with high mortality should prompt surveillance.

## Introduction

*Klebsiella pneumoniae* (KP) is one of the most common bacterial pathogens that causes community or nosocomial acquired infections, such as pneumonia, urinary tract and surgical site infections, bloodstream infections (BSIs), and hepatobiliary infections (Paczosa and Mecsas, 2016). The management of infections due to KP has been complicated by the emergence of antimicrobial resistance and hypervirulent *K. pneumoniae* (HvKp). In particular, disappointing clinical outcomes have been observed in patients with KP BSIs when accompanied by hypervirulent phenotype or multidrug-resistant organisms. The drugs of β-lactams are recognized as the most common use antimicrobial agents due to their high efficiency and lower toxicity. However, carbapenem resistance in KP is a growing problem worldwide, and in China, as China Antimicrobial Surveillance Network had shown, resistance to carbapenems in KP has increased from 2.9% in 2005 to 25% in 2018 (Zheng et al., 2019; Hu et al., 2020). More importantly, carbapenem-resistant HvKp have emerged in clinical settings, although most KP of hypervirulent and antimicrobial-resistant populations was largely non-overlapping (Bialek-Davenet et al., 2014; Zhang et al., 2016; Gu et al., 2018). The prevalence of such organisms poses a significant challenge for clinicians worldwide and often leads to the failure of clinical treatment (Lin et al., 2010; Mohammad Ali Tabrizi et al., 2018). Previous studies have indicated that mortality rates of KP BSIs range from 16% to 40% depending on the conditions of patients and characteristics of bacterial isolates as well as administrations of appropriate antibiotic therapy (Shon et al., 2013; Kohler et al., 2017; Xu et al., 2017; Kim et al., 2019). For the bacterial pathogenic traits, evaluating the elements of virulence factors and antimicrobial susceptibility are crucial in the improvements of clinical practice for managing KP BSI patients.

Several classical traits have been associated with HvKp as compared to classical KP, such as a hypermucoviscous phenotype, predominance of K1 and K2 capsule type, and carriage of multiple virulence genes (e.g., *rmpA*, *rmpA2*, *iroBCDN*, *iucABCD*, and *iutA*) (Lee et al., 2017; Russo et al., 2018). The risk factors for HvKp infections in patients with KP BSIs have been widely studied (Li et al., 2018; Harada et al., 2019). Impacts of virulence factors on mortality of KP BSIs in human patients have not been fully revealed, although fatal outcomes of HvKp infections have been observed in *in vivo* mouse infection models (Lery et al., 2014; Russo et al., 2015).

Most previous studies have reported that severity of underlying disease, intensive care unit (ICU) stay at infection onset, infection with extended-spectrum β-lactamase (ESBL)-producing or carbapenem-resistant KP (CRKP), and delayed administrations of appropriate therapy are the common risk factors for mortality in patients with KP BSIs (Viale et al., 2013). Among the pathogenic factors, studies focusing on the virulence factors related to mortality in patients with KP BSI are scarce (Kim et al., 2019; Namikawa et al., 2019). Kim et al. indicated that carriage of the *pks* gene cluster was a relevant marker of early mortality using multivariate Cox hazards modeling (Kim et al., 2019). Another study of multivariate analysis showed that *iutA* was an independent predictor associated with increased 30-day mortality in patients with KP BSIs (Namikawa et al., 2019). Notably, the virulence genes detected in previous studies were assessed by PCR, which may not fully address the clinically important genes influencing KP pathogenicity and virulence. The increasing availability of bacterial whole-genome sequencing (WGS) allows for the practical identification of virulence factors in bacteria, including *K. pneumoniae* (Holt et al., 2015). WGS and bioinformatics analyses combined with clinical characteristics can fully identify associations between bacterial accessory genome and mortality in patients with BSIs.

The aims of our study were to (i) describe the molecular epidemiology of KP in BSI patients at a tertiary hospital and (ii) evaluate the clinical variables and genetic backgrounds associated with mortality in patients with KP BSIs.

## Methods

### Study Design

This retrospective cohort study included all patients with KP BSI at a 1200-bed tertiary teaching hospital between January 1, 2016, and December 31, 2019, in Zhejiang Province, China. BSI was defined as one or more positive blood cultures with concomitant symptoms of infection, according to established criteria (Garner et al., 1988). Only the first episode of BSI was included, and the remaining sequential isolates were discarded. The detailed inclusion criteria were as follows: (a) patients aged ≥18 years; (b) hospitalization with a complete clinical data set; (c) a blood culture positive for KP and the sample preserved in our laboratory; and (d) clinical manifestations of infection. Outpatients, patients with incomplete data, and those lacking KP isolates were excluded. This study was approved by the Ethics Committee of Lihuili Hospital, Ningbo Medical Center (no. KY2019PJ028).

### Definitions and Patient Assessment

The probable infectious source was determined using the CDC/National Healthcare Safety Network surveillance definitions (Centers for Disease Control and Prevention). Corticosteroid therapy was defined as the administration of >20 mg/day prednisone (or its equivalent) for a period of ≥7 days. Antimicrobial drug exposure was defined as the use of antibiotics for more than 48 h within 90 days prior to the onset of BSI. Empirical antimicrobial treatment was used to treat suspected KP BSI without *in vitro* antimicrobial susceptibility information, while definitive antimicrobial treatment was revised based on the *in vitro* antimicrobial susceptibility results. Antimicrobial therapy was determined to be appropriate when the treatment regimen included antibiotics active against pathogens *in vitro*. The final outcome was determined as survival and all-cause death at 30 days after the date of BSI onset.

### Data Collection

The demographic and clinical information of the enrolled patients, including age, sex, underlying disease, according to the age-adjusted Charlson Comorbidity Index (aCCI), possible sources of BSI, antibiotic regimen, patient outcomes, and other relevant information, were retrieved from the electronic medical records system (Charlson et al., 1994). Moreover, the Acute Physiology and Chronic Health Evaluation (APACHE) II and Pitt bacteremia scores calculated at the time of BSI onset were used to assess illness severity (Knaus et al., 1985).

### Bacterial Isolates and Antimicrobial Resistance Susceptibility

The string test was performed with a standard bacteriologic loop to evaluate hypermucoviscosity, and the formation of viscous strings >5 mm in length was considered positive (Fang et al., 2004). The virulence genes *iucA*, *iroB*, *peg-344*, *rmpA*, and *rmpA2* were assessed using PCR (Russo et al., 2018). The primers used in this study are listed in **Supplementary Table S1**.

The minimum inhibitory concentrations (MICs) of ceftriaxone (CRO), cefepime (FEP), cefoperazone-sulbactam (1:1) (CFZ/SBT), piperacillin-tazobactam (PIP/TZP), imipenem (IPM), meropenem (MEM), levofloxacin (LEV), fosfomycin (FOS), and amikacin (AMK) against the collected strains were determined using the agar dilution method, according to the Clinical and Laboratory Standards Institute (CLSI) (Clinical and Laboratory Standards Institute). For colistin (CST) and tigecycline (TGC), the broth microdilution method was used.

### Whole-Genome Sequencing and Bioinformatics Analysis

To characterize the genetic features of BSI isolates, WGS was performed using the Illumina HiSeq platform (Illumina, San Diego, CA, USA). Sequencing data were assembled using SPAdes v. 3.15.0. The antimicrobial resistance genes were identified using the resistance gene identifier through the Comprehensive Antibiotic Research Database. The virulence genes were annotated using the Diamond software through the Virulence Factors Database (VFDB). KP surface polysaccharide locus typing was annotated using Kaptive (Wyres et al., 2016). Multi-locus sequence typing (MLST) was performed using the MLST software (https://github.com/tseemann/mlst), which incorporates the components of the PubMLST database. The kSNP program based on the k-mer analysis was used to identify the core genomic single-nucleotide polymorphisms in the WGS data. Kchooser was used to evaluate the optimal value of k-mer before kSNP. The output file of the maximum likelihood tree was generated using the iTOL (https://itol.embl.de/). As the strains of R5 and R18 (Sequence Read Archive database with SRP141269) were regarded as carbapenem-resistant HvKp *via* bacteriological test, neutrophil killing assay, and *Galleria mellonella* infection model in a previous study, comparative genomic analysis on the phylogenies and virulence of ST11 KP was conducted using by kSNP and Roary (Yang et al., 2020). The phylogenies of the ST11 strains were performed by kSNP software based on the core genome SNPs. The Roary software was used to calculate the pan-genome (Page et al., 2015). The virulence factors were identified by blasting the VFDB database using Diamond (Buchfink et al., 2021).

### Statistical Analysis

Analyses were conducted using the R software (version 4.0.2; http://www.R-project.org/). Continuous variables are expressed as the mean ± standard deviation (assessed using Student’s *t*-test) or as the median (range) (evaluated using the Wilcoxon rank-sum test) when the distribution was not normal. Categorical data were expressed as frequency distributions, and the chi-square test or Fisher’s exact test was used to determine the distribution when appropriate. The clinically important variables, antimicrobial resistance, and virulence determinants (*p* < 0.1) in univariate analysis were then selected into a logistic regression model for multivariate analysis to evaluate the risk factors for KP BSI mortality. Notably, the above variables were determined using the variance inflation factor (VIF) and the eigenmatrix method to detect multicollinearity before logistic regression analysis. Binary logistic regression (step: Akaike information criterion [AIC], direction: backward and forward) was used to identify independent predictors of 30-day mortality. The strength of associations was determined by calculating the odds ratio (OR) and 95% confidence intervals (CIs). Two-tailed tests were used to determine the statistical significance. The survival distribution function was estimated using the Kaplan–Meier product limit method. Nonparametric (log-rank and Wilcoxon) tests were used to compare survival functions between the groups. In all analyses, a *p*-value of ≤0.05 was considered significant.

### Nucleotide Sequence Accession Numbers

The whole-genome sequences described in this paper have been deposited in the National Microbiology Data Center under the accession numbers NMDC60014864-NMDC60014942.

## Results

### Demographic and Clinical Characteristics

In total, 92 unique cases of KP BSI during the 4-year study period were identified. Of these, five patients were excluded because of missing KP isolates, and eight cases were excluded owing to early death. Finally, 79 patients (48 males and 31 females) with laboratory-confirmed KPs were enrolled. The clinical characteristics of KP BSI between non-survivors and survivors are summarized in **Table 1** and **Supplementary Table S2**. The median age of all patients was 67 years (interquartile range, 59.0–77.5). Hepatobiliary disease (27.8%) was the most prevalent comorbidity, followed by diabetes mellitus (21.5%) and malignancy (15.2%). The median age-aCCI was 3 (IQR, 2–4). The most common probable source of infection was pneumonia (43.0%), followed by biliary tract infections (13.9%) and liver abscesses (12.7%). The median time to the onset of KP BSI was 8 days (IQR, 1–23.5). Almost half of the patients resided in the ICU within 90 days prior to the onset of BSI. The median APACHE II and Pitt bacteremia scores at the time of the initial blood culture were 17 (IQR, 13–26) and 2 (IQR, 1–6), respectively. In addition, the 30-day mortality rate of patients infected with KP BSI was 30.4%.

**Table 1:** Clinical characteristics and laboratory findings of non-survivor and survivor patients with *K. pneumoniae* bloodstream infection.

Variables	No. of patients (%) or parameter value (mean ± SD)			*p*-value
	Overall (*n* = 79)	Non-survivors (*n* = 24)	Survivors (*n* = 55)	
Age, years (IQR)	67.00 (59.00–77.50)	64.00 (56.75–73.75)	70.00 (60.00–79.00)	0.224
Male	48 (60.8)	17 (70.8)	31 (56.4)	0.337
Pre-existing medical conditions				
Diabetes mellitus	17 (21.5)	3 (12.5)	14 (25.5)	0.322
Pulmonary disease	10 (12.7)	2 (8.3)	8 (14.5)	0.692
Cardiovascular disease	20 (25.3)	4 (16.7)	16 (29.1)	0.375
Hepatobiliary disease	22 (27.8)	2 (8.3)	20 (36.4)	**0.022**
Median aCCI (IQR)	3.00 (2.00–4.00)	3.00 (1.75–4.25)	3.00 (2.00–4.00)	0.991
Prior ICU stay, day (IQR)	37 (46.8)	18 (75.0)	19 (34.5)	**0.002**
Invasive procedure or devices	48 (60.8)	19 (79.2)	29 (52.7)	**0.05**
Mechanical ventilation	31 (39.2)	18 (75.0)	13 (23.6)	**<0.001**
Central venous catheterization	37 (46.8)	19 (79.2)	18 (32.7)	**<0.001**
Urinary catheterization	43 (54.4)	19 (79.2)	24 (43.6)	**0.008**
Stomach tube catheterization	44 (55.7)	19 (79.2)	25 (45.5)	**0.011**
Prior hemodialysis	6 (7.6)	6 (25.0)	0 (0.0)	**0.001**
Prior corticosteroid use	21 (26.6)	11 (45.8)	10 (18.2)	**0.023**
Corticosteroid use after BSI	20 (25.3)	10 (41.7)	10 (18.2)	0.054
Prior receipt of antibiotics in the 30 days prior to BSI				
BLBLIs	23 (29.1)	13 (54.2)	10 (18.2)	**0.03**
Carbapenem	27 (34.2)	12 (50.0)	15 (27.3)	0.089
Tigecycline	6 (7.6)	4 (16.7)	2 (3.6)	0.121
Laboratory examination				
BUN	8.57 (5.38)	12.38 (6.83)	6.91 (3.55)	**<0.001**
CRP	110.42 (73.91)	115.50 (64.28)	108.21 (78.19)	0.69
Severity of illness at time of BSI				
APACHE II score	17.00 (13.00–26.00)	27.50 (22.00–30.25)	14.00 (11.00–18.00)	**<0.001**
PITT	2.00 (1.00–6.00)	6.50 (4.00–7.25)	1.00 (1.00–3.00)	**<0.001**
Septic shock	17 (21.5)	11 (45.8)	6 (10.9)	**0.001**
Antimicrobial after BSI				
Empiric treatment of BLBLIs	26 (32.9)	13 (54.2)	13 (23.6)	**0.017**
Empiric treatment of carbapenems	41 (51.9)	10 (41.7)	31 (56.4)	0.338
Appropriate empirical treatment	54 (68.4)	12 (50.0)	42 (76.4)	**0.04**

APACHE, Acute Physiology, and Chronic Health Evaluation; aCCI, age-adjusted Charlson Comorbidity Index; BSI, bloodstream infection; BUN, blood urea nitrogen; ICU, intensive care unit; IQR, interquartile range; BLBLI, beta-lactam-beta-lactamase inhibitor. Bold formatting indicates statistical significance.

### Bacterial Characteristics

**Figure 1:**
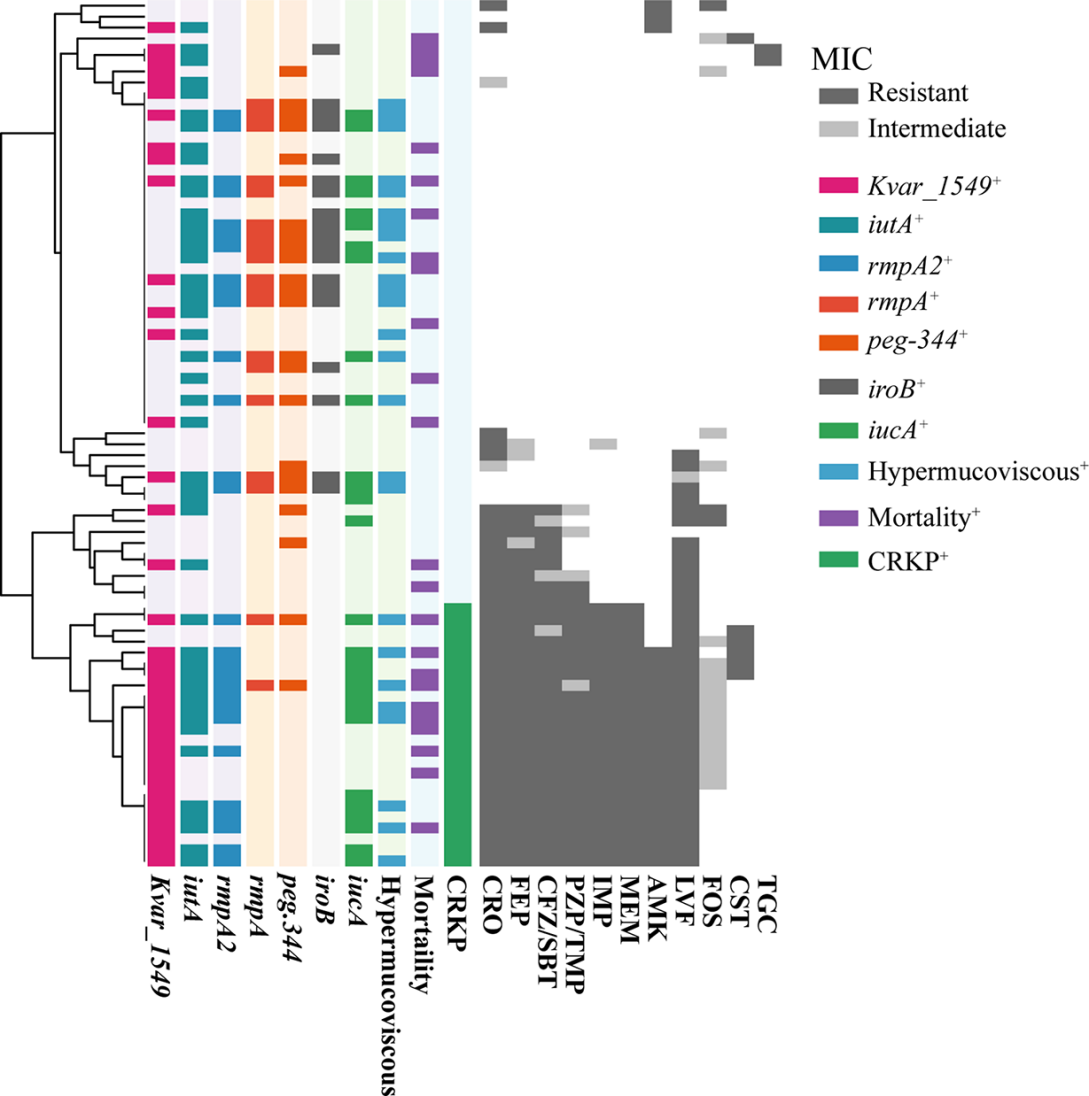
The MIC distribution of all antibiotics against *K. pneumoniae* isolated from bloodstream infection. MICs were using agar dilution methods for all antibiotics except for colistin and tigecycline, for which broth microdilution was used. Results were interpreted using the CLSI guidelines. Dark gray indicates resistance, light gray indicates mediation, and white indicates sensitivity. The tree on the left side was reconstructed by hierarchical clustering of MIC results using the Euclidean agglomeration method. The colored blocks in the middle represent the presence of virulence genes/phenotype, and white blocks represent absence. CRO, Ceftriaxone; FEP, Cefepime; CFZ/SBT, Ceftolozane-sulbactam; PZP/TMP, Piperacillin-tazobactam; IMP, Imipenem; MEM, Meropenem; AMK, Amikacin; LVF, Levofloxacin; FOS, Fosfomycin; CST, Colistin; TGC, Tigecycline. Mortality, 30-day mortality. CRKP, carbapenem-resistant *K. pneumoniae*.

The MIC results demonstrated that MDRKP and CRKP isolates, especially the latter, exhibited multidrug resistance to most classes of antimicrobial agents, except FOS, TGC, and CST (**Figure 1** and **Supplementary Table S3**). CRKP was more likely to be observed in patients with prior admission to the ICU, and higher mortality was observed in the CRKP group (**Figure 1** and **Table 2**). Regarding antimicrobial resistance genes, 114 resistance genes were detected in our study, including *bla*_CTX-M_, *bla*_SHV_, *bla*_TEM_, *bla*_KPC-2_, *bla*_OXA-1_, *aac(3)-IId*, *qnr*, *tet*, *fosA5*, *sul*, and *aadA* (**Table 2** and **Supplementary Table S4**). Among them, *bla_SHV_* (89.9%, *n* = 71) and *fosA6* (91.1%, *n* = 72) were the most commonly identified resistance genes in the tested isolates. The most prevalent *bla*_CTX-M_ gene was *bla*_CTX-M-65_ (22.8%, *n* = 18), followed by *bla*_CTX-M-14_ (6.3%, *n* = 5), and *bla*_CTX-M-15_ (5.1%, *n* = 4). The *bla*_KPC-2_ gene, which is responsible for carbapenem resistance, was also observed in 24 cases.

**Table 2:** Bacterial characteristics of the non-survivor and survivor patients with *K. pneumoniae* bloodstream infection.

Variables	No. of patients (%)			*p*-value
	Overall (*n* = 79)	Non-survivors (*n* = 24)	Survivors (*n* = 55)	
MDRKP	36 (45.6)	12 (50.0)	24 (43.6)	0.782
CRKP	24 (30.4)	10 (41.7)	14 (25.3)	0.182
Hypermucoviscous phenotype	25 (31.6)	16 (29.1)	9 (37.5)	0.634
**Resistance genes**				
Cephalosporins				
CTX-M	33 (41.8)	23 (41.8)	10 (41.7)	1
* CTX-M-65*	18 (22.8)	10 (18.2)	8 (33.3)	0.236
SHV	78 (89.7)	25 (80.6)	53 (94.6)	0.092
* SHV-1*	5 (5.7)	1 (3.2)	4 (7.1)	0.786
Aminoglycosides				
AAC	21 (24.1)	7 (22.6)	14 (25.0)	1
* AAC(3)-IId (%)*	12 (13.8)	3 (9.7)	9 (16.1)	0.614
Carbapenems				
* KPC-2*	24 (27.6)	9 (29.0)	15 (26.8)	1
Tetracycline				
tet	24 (27.6)	8 (25.8)	16 (28.6)	0.979
* tet(A) (%)*	19 (21.8)	6 (19.4)	13 (23.2)	0.884
**Virulence genes**				
Mucoviscosity				
* iucA*	31 (35.6)	13 (41.9)	18 (32.1)	0.497
* iroB*	21 (24.1)	6 (19.4)	14 (25.0)	0.614
* peg-344*	25 (28.7)	7 (22.6)	18 (32.1)	0.486
* rmpA*	20 (23.0)	5 (16.1)	15 (26.8)	0.387
* rmpA2*	31 (35.6)	12 (38.7)	19 (33.9)	0.832
Biosynthesis of lipopolysaccharide				
* kfoC*	24 (30.4)	21 (38.2)	3 (12.5)	**0.044**
Capsule				
* Kvar_1549*	38 (48.1)	21 (38.2)	17 (70.8)	**0.015**
Iron uptake and transport				
* iutA*	45 (57.0)	27 (49.1)	18 (75.0)	0.058
Fimbriae				
* mrkB*	76 (96.2)	52 (94.5)	24 (100.0)	0.599
Secretion				
* pulC*	8 (10.1)	3 (5.5)	5 (20.8)	0.093
* pulM*	10 (12.7)	4 (7.3)	6 (25.0)	0.07

MDRKP, multidrug-resistant K. pneumoniae; CRKP, carbapenem-resistant K. pneumoniae; BSI, bloodstream infection. Bold formatting indicates statistical significance.

**Figure 2:**
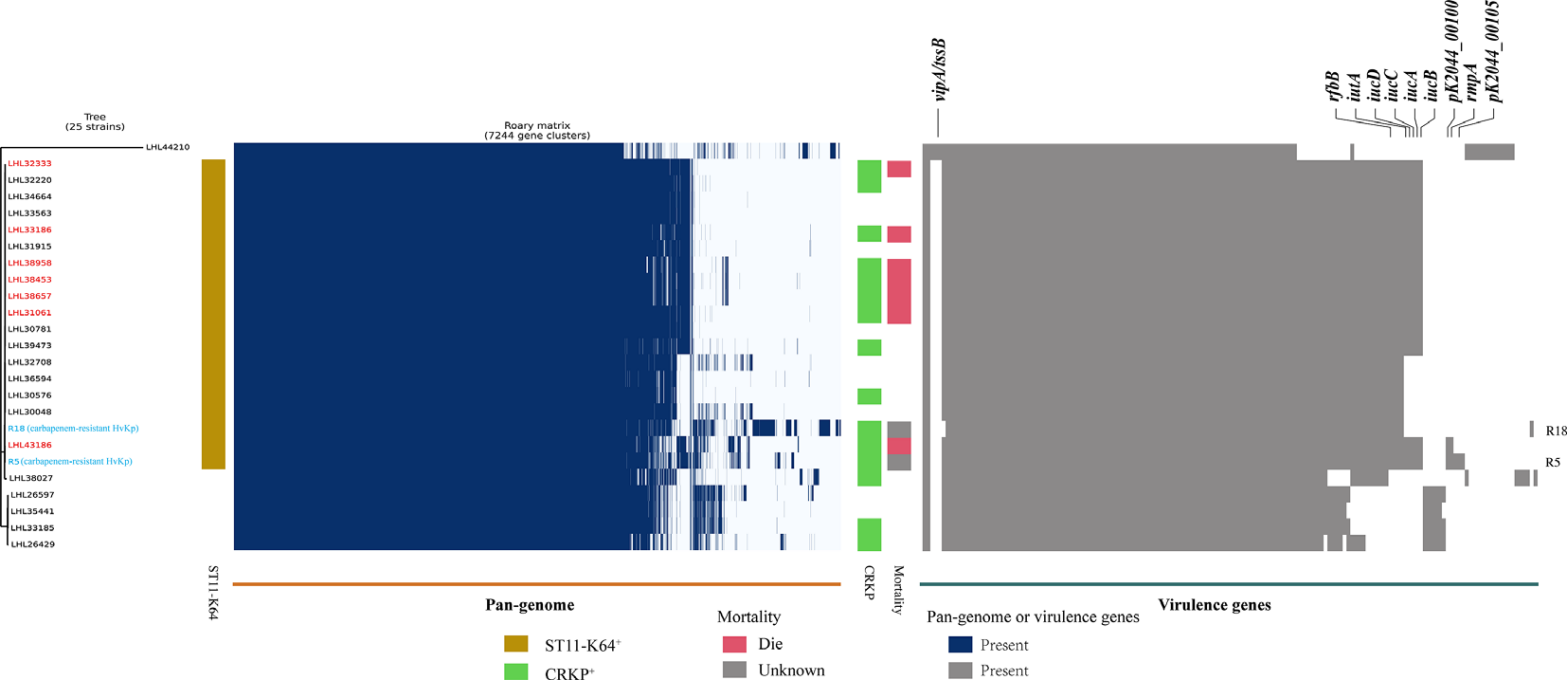
Phylogenetic analysis, pan-genome analyses, and virulence genes of ST11 *K. pneumoniae* strains. Phylogenetic analysis, pan-genome, and virulence genes are shown on the left, middle, and right, respectively. Phylogenetic analysis was performed by kSNP software based on the core genome SNPs. Strains are color-coded on the tree. Two carbapenem-resistant hypervirulent *K. pneumoniae* R5 and R18 (marked with blue) were selected from the NCBI SRA database as references. Patients with a fatal outcome were marked with red color. Each row of the heatmap indicated a strain, and each column represented a gene. Colored blocks represent the presence of factors, and white blocks represent absence. The mortality bar of R5 and R18 strains were marked with gray color as unknown outcomes.

A positive string test was observed in one-third of the isolates (31.6%, *n* = 25), and most of them harbored at least one of the *rmpA*/*rmpA2*/*iucA*/*iroB*/*peg-344* genes (5/5, *n* = 7; 4/5, *n* = 9; 3/5, *n* = 1; 2/5, *n* = 7; 1/5, *n* = 1; 0/5, *n* = 1) (**Table 2** and **Figure 1**). The most prevalent virulence genes related to mucoviscosity were *iucA* (35.6%, *n* = 31) and *rmpA2* (35.6%, *n* = 31), followed by *peg-344* (28.7%, *n* = 25) and *iroB* (24.1%, *n* = 21) (**Table 2**). To better understand the genetic basis of KP strains, MLSTs and serotypes were further analyzed. For K1 and K2 isolates, hypermucoviscous phenotypes were frequently detected (78.6%, 11/14) while resistance genes were scarce. MLST results showed that ST11 was the most commonly identified ST (30.4%, *n* = 24), followed by ST25 (6.3%, *n* = 5) and ST23 (5.1%, *n* = 4) (**Supplementary Table S5**). The most prevalent capsular type in our strains was K64 (25.3%, *n* = 20), followed by K2 (10.1%, *n* = 8) and K1 (7.6%, *n* = 6). Notably, most of the K64 isolates co-carried genes for beta-lactamases (*bla*_CTX-M_, 90%, *n* = 18) and carbapenemases (*bla*_KPC-2_, 95%, *n* = 19). All the ST11-K64 strains (*n* = 17) were related to the acquisition of *bla*_KPC-2_ gene, and most co-harbored *rmpA2* (76.5%, 13/17), *iucA* (70.6%, 12/17), *iutA* (76.5%, 13/17), and *Kvar_1549* (100%, 17/17) genes. Furthermore, all the ST11-K64 strains in our study were clustered together with ST11-K64 strains R5 and R18, which were confirmed as carbapenem-resistant HvKp in a previous study. ST11-K64 and ST11-non-K64 strains appear to have evolved separately into two different branches. For virulence factors, more virulence genes, including *rmpA*, *iutA*, *iucA*, *iucB*, *iucC*, and *iucD*, were observed in ST11-K64 strains compared with ST11-non-K64 strains (**Figure 2**). The mortality rate of ST11-K64 strains was 47.2% (7/17) compared with 0% (0/6) of ST11-non-K64 strains.

**Figure 3:**
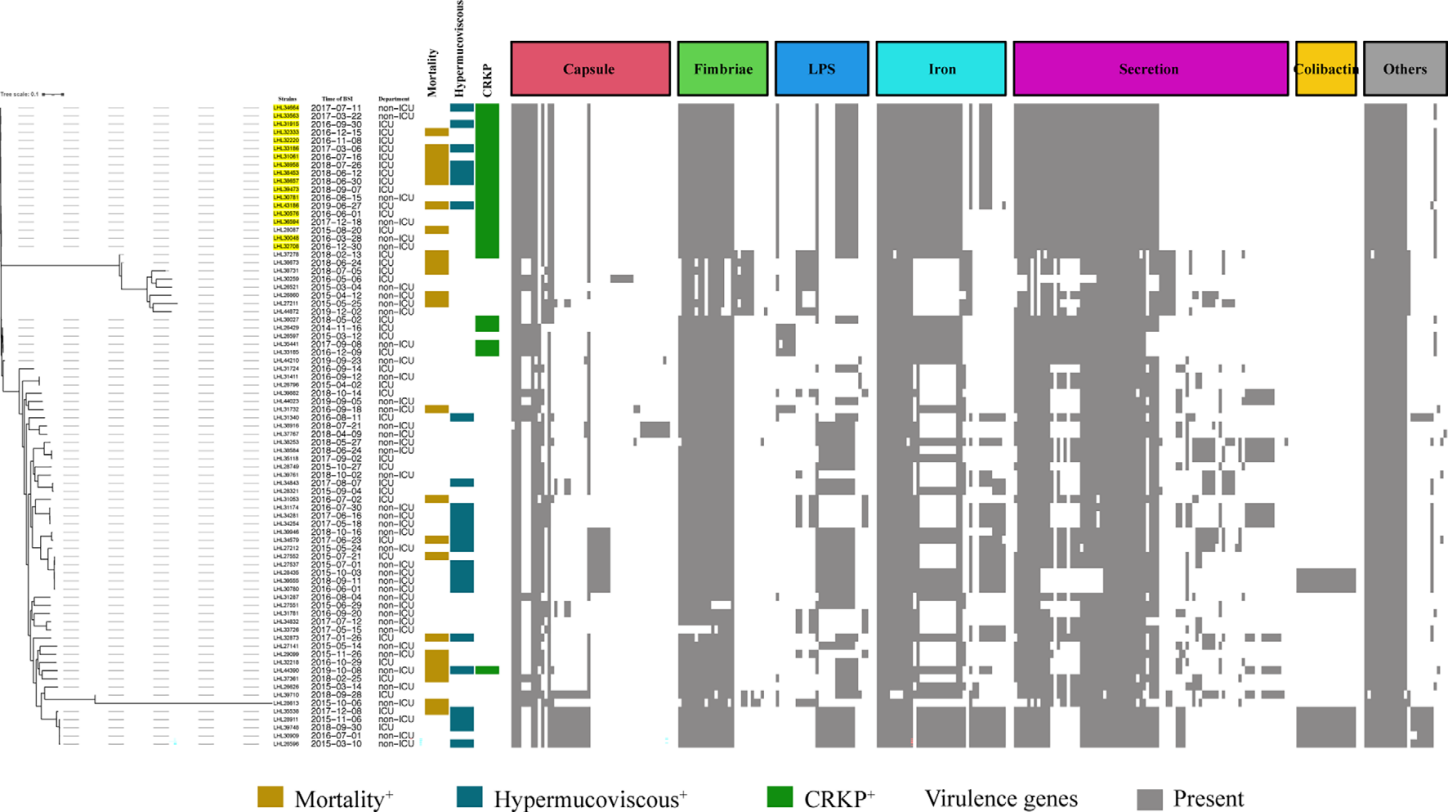
Evolutionary relationships, virulence genes, and distribution of *K. pneumoniae* strains. Evolutionary relationships and virulence genes are shown on the left and right, respectively. ST11-K64 strains are color-coded on the tree (yellow). Each row of the heatmap indicated a strain, and each column represented a virulence gene that belonged to the indicated functional clusters shown at the top. Colored blocks represent the presence of genes, and white blocks represent absence.

The BLAST results identified 268 virulence genes in the tested KP, including biosynthesis of lipopolysaccharide, iron uptake and transport, capsule, fimbriae, and secretion (**Supplementary Table S4**). As shown in **Figure 3**, the heatmap of virulence traits showed that secretion-associated genes and genes related to iron uptake and transport and fimbriae were frequently detected in our strains. It should be noted that relatively more virulence genes in iron uptake and secretion were possessed by ST11-K64 strains than by some carbapenem-susceptible KP strains (**Figure 3**).

### Risk Factors for 30-Day Mortality in Patients With KP BSI

Of the 79 patients enrolled, 55 (69.6%) were classified as survivors and 24 (30.4%) were classified as non-survivors. In the univariate logistic analysis modeling by host factors, prior admission to the ICU, invasive procedures or devices, increased Pitt and APACHE II score at onset of BSI, corticosteroid use before or after BSI, and septic shock exhibited a significant association with increased 30-day mortality. Conversely, the adequacy of empirical antimicrobial treatment and empirical treatment with β-lactam-β-lactamase inhibitor (BLBLI) was associated with decreased 30-day mortality.

Regarding antimicrobial susceptibility, neither CRKP/MDRKP nor the hypermucoviscous phenotype showed a positive association with 30-day mortality. Accordingly, none of the resistance genes were significantly associated with increased 30-day mortality. For virulence traits, several virulence genes were associated with increased 30-day mortality. Notably, the tested strains carried *pulC*, and all of them co-harbored *KP1_2101*, *A79E_1988*, *N559_2629*, *Kvar_1936*, *Kvar_0795*, and *Kvar_2690*. Similar results were found for *pulM* and *kfoc* (**Supplementary Table S4**). A detailed univariate logistic analysis is shown in **Supplementary Table S4**.

Because of multicollinearity among the clinical and interconnected nature of the bacterial characteristics, the eigenmatrix method and VIF were conducted before the variables were selected for logistic modeling. Multivariate logistic modeling was performed using four separate sets of adjusted analyses. The variables of the host factors were included in all sets. The increased APACHE II score and septic shock were strongly associated with increased 30-day mortality in all four sets. For the virulence traits, *iutA*, *Kvar_1549*, *pulM*, and *pulC* were conducted using four separate sets because of multicollinearity among them. *iutA* (OR, 1.17; 95% CI, 0.97–1.37; *p* = 0.093), *Kvar_1549* (OR, 1.16; 95% CI, 0.96–1.35; *p* = 0.102), *pulM* (OR, 1.31; 95% CI, 1.02–1.69; *p* = 0.038), and *pulC* (OR, 1.31; 95% CI, 0.99–1.73; *p* = 0.066) were independent predictors of the 30-day mortality of KP BSI (**Table 3**).

**Table 3:** Multivariate analysis of predictors associated with the 30-day mortality of *K. pneumoniae* bloodstream infection.

Predictor	Set 1^a^		Set 2		Set 3		Set 4	
	OR (95% CI)	*p*-value	OR (95% CI)	*p*-value	OR (95% CI)	*p*-value	OR (95% CI)	*p*-value
APACHE II score	1.48 (1.23–1.79)	**<0.001**	1.49 (1.24–1.80)	**<0.001**	1.43 (1.18–1.73)	**<0.001**	1.44 (1.18–1.74)	**<0.001**
Septic shock	1.23 (0.99–1.51)	0.06	1.21 (0.98–1.50)	0.076	1.31 (1.06–1.61)	**0.014**	1.29 (1.05–1.59)	**0.019**
*iutA*	1.17 (0.98–1.37)	0.085	–	–	–	–	–	–
*Kvar_1549*	–	–	1.16 (0.97–1.35)	0.082	–	–	–	–
*pulM*	–	–	–	–	1.31 (1.02–1.69)^b^	**0.038**	–	–
*pulC*	–	–	–	–	–	–	1.31 (0.99–1.73)^c^	0.066

a Because the variance inflation factor and the eigenmatrix method showed multicollinearity among bacterial factors, multivariate logistic modeling was performed using four separate sets of adjusted analyses to assist in the interpretation of the effect of each bacterial factor. Variables of host factors were included in all four sets of analyses.

b The strain co-harbored pulM, fliY, Kvar_1938, Kvar_0779, mrkF, and KPK_0838 genes simultaneously.

c The strain co-harbored pulC, KPK_2690, Kvar_1936, Kvar_0771, and Kvar_0795 genes simultaneously. APACHE, Acute Physiology and Chronic Health Evaluation; OR, odds ratio; CI, confidence interval. Bold formatting indicates statistical significance.

### Risk Factors for 30-Day Mortality in Patients With MDRKP BSI

Subgroup analysis was performed according to the MDR phenotype. In total, 36 patients were enrolled, including 12 non-survivors and 24 survivors. The univariate logistic analysis indicated that increased Pitt and APACHE II scores at the onset of BSI, hypermucoviscous phenotype, mechanical ventilation, septic shock, *iutA*, *rmpA2*, and *Kvar_1549* were associated with increased 30-day mortality (**Supplementary Tables S6, S7**). Multivariate analysis revealed that increased APACHE II score, septic shock, and *iutA* (OR, 1.46; 95% CI, 1.11–1.81, *p* = 0.002) in set 1 and APACHE II score, septic shock, hypermucoviscous phenotype (OR, 1.24; 95% CI, 0.92–1.68, *p* = 0.168), and *Kvar_1549* (OR, 1.31; 95% CI, 1.02–1.69, *p* = 0.043) in set 2 increased the 30-day mortality of MDRKP BSI (**Table 4**).

**Table 4:** Multivariate analysis of predictors associated with the 30-day mortality of MDRKP bloodstream infection.

Predictor	Set 1^a^		Set 2	
	OR (95% CI)	*p*-value	OR (95% CI)	*p*-value
APACHE II score	1.56 (1.25–1.93)	**<0.001**	1.56 (1.25–1.96)	**<0.001**
Septic shock	1.32 (1.04–1.67)	**0.029**	1.28 (1.00–1.65)	**0.056**
Hypermucoviscous phenotype	–	–	1.24 (0.92–1.68)	**0.162**
*iutA*	1.46 (1.11–1.81)	**0.002**	–	–
*Kvar_1549*	–	–	1.31 (1.02–1.69)	**0.043**

a Because the variance inflation factor and the eigenmatrix method showed multicollinearity among bacterial factors, multivariate logistic modeling was performed using two separate sets of adjusted analyses to assist in the interpretation of the effect of each bacterial factor. Variables of host factors were included in all two sets of analyses. APACHE, Acute Physiology and Chronic Health Evaluation; MDRKP, multidrug-resistant K. pneumoniae; BSI, bloodstream infection. Bold formatting indicates statistical significance.

**Figure 4:**
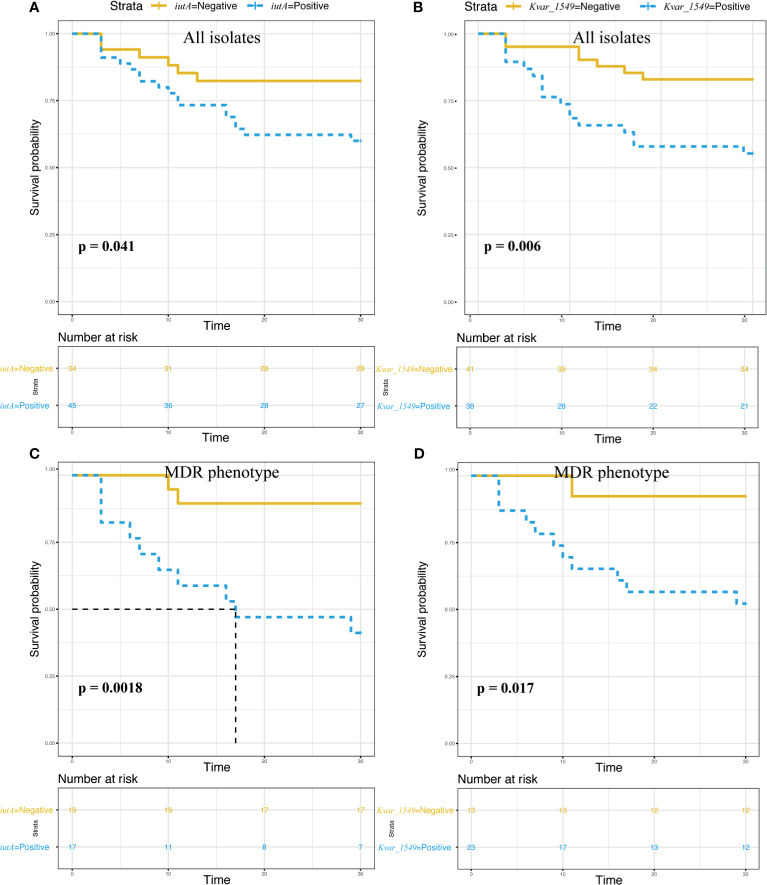
Survival analysis according to the carriage of the *iutA* and *Kvar_1549* genes. Statistical significance was used by the log-rank test. **(A, C)** *iutA* gene in all the *K. pneumoniae* and MDR groups, respectively. **(B, D)** *Kvar_1549* gene in all the *K. pneumoniae* and MDR groups, respectively.

The survival curves showed a significant impact on 30-day mortality for *iutA* (75.0% *vs.* 25.0%, *p* = 0.041) and *Kvar_1549* (70.8% *vs.* 29.2%, *p* = 0.006) (**Figures 4A, B**). Similar results were observed in the subgroup of MDRKP BSI carrying *iutA* (83.3% *vs.* 16.7%, *p* = 0.002) or *Kvar_1549* (91.7% *vs.* 8.3%, *p* = 0.017) genes (**Figures 4C, D**).

## Discussion

This study highlighted that *iutA* and *Kvar_1549* were associated with increased 30-day mortality in KP BSI. Of note, higher mortality was observed when *iutA* and *Kvar_1549* carriage by causative strains was accompanied by MDR. Some tested strains in the present study co-harbored resistance determinants and hypervirulence genes. Previous studies have indicated that hypervirulent and MDR strains evolved separately in different clonal groups, and MDRKP had lower virulence, whereas HvKp was sensitive to most antibiotics (Russo and Marr, 2019). The presence of either drug resistance or virulence genes in bacterial isolates may lead to treatment failure, severe life-threatening infections, and subsequent prolongation of the illness (Tumbarello et al., 2012; Viale et al., 2013; Russo and Marr, 2019). The integration of these factors together may further complicate clinical practice. MDR or carbapenem-resistant HvKp isolated from different clinical settings result in fatality and have begun to spread in China (Zhang et al., 2015; Gu et al., 2018; Huang et al., 2018; Zheng et al., 2020). Recently, a multi-center epidemiological and comparative genomic analysis indicated that the ST11-K64 hypervirulent CRKP, isolated from bacterial liver abscesses, was simultaneously MDR and hypervirulent using comprehensive test combinations including string test, human neutrophil killing assay, and *G. mellonella* infection model (Yang et al., 2020). We identified 17 ST11-K64 isolates, which were all related to the acquisition of *bla*_KPC-2_ gene, and most co-harbored *rmpA2* (76.5%, 13/17), *iucA* (70.6%, 12/17), *iutA* (76.5%, 13/17), and *Kvar_1549* (100%, 17/17) genes in our study. Comparative genome analysis showed that the ST11-K64 strains were phylogenetically closely related to carbapenem-resistant HvKp strains R5 and R18. More virulence factors were observed in the group of ST11-K64 carbapenem-resistant HvKp, which may account for its high mortality (47.2%, 7/17) rate in patients infected with this strain. In addition, these strains exhibited sustained survival during the 4-year study period, especially in the ICU department, indicating that they may chronically colonize the surrounding environment. This study provides clinical evidence of the hypervirulence of ST11-K64 carbapenem-resistant HvKp. Therefore, urgent control measures are required for its dissemination.

Capsule polysaccharides (CPS) are a major virulence factor in most isolates because of their ability to evade phagocytosis and complement-mediated killing and further inhibit complement activation of the host (Fang et al., 2004; Cozzone, 2005). Hypervirulence is associated with the overproduction of CPS, and in the absence of related virulence genes, its virulence is reduced or abolished (Favre-Bonte et al., 1999; Wyres et al., 2016). Shon et al. (2013) assumed that the high level of virulence was due to an increased expression of CPS combined with increased efficiency of iron acquisition and other traits. *Kvar_1549*, identified in our logistic model, was found to be associated with CPS. Increased mortality was observed in patients infected with these strains, with borderline differences in all KP and significant differences in the MDR phenotype. The protein encoded by *Kvar_1549* is a type 2 phosphatidic acid phosphatase-like (PAP2) protein, which controls various physiological functions, such as CPS and lipopolysaccharide synthesis. However, to date, no cases of *Kvar_1549* have been associated with increased 30-day mortality in patients with KP BSI. Zhao et al. (2019) indicated that LpxE, a PAP2 encoded protein, participates in multiple layers of biogenesis of the gram-negative bacterial envelope and increases antibiotic resistance and bacterial virulence. Thus, *Kvar_1549* may be responsible for the formation of CPS, leading to enhanced virulence. Notably, the prevalence of *Kvar_1549* in the tested strains was 48.1%. Continued studies regarding the role of *Kvar_1549* should be performed using molecular analysis and applicable infection models because of its high prevalence in KP isolates.

*iutA*, the fifth gene of the aerobactin operon, encodes a specific outer membrane receptor protein for iron uptake. The ability to acquire iron is essential for bacterial growth and replication owing to its role as a cofactor for several enzymes, such as those involved in electron transport and amino acid and DNA biosynthesis (Wandersman and Stojiljkovic, 2000). This iron uptake system plays a crucial role in the progression of infection. Vargas et al. (2019) indicated that *iutA* promotes biofilm formation. Tang et al. (2010) showed that *iutA* is an independent pathogenicity factor for abscess formation. An *in vitro* study revealed that the expression of *iutA* was upregulated in pathogenic bacteria under iron-depleted conditions (Torres et al., 2012). Further tests revealed that the *iutA* mutant was outcompeted by the wild-type strain in the murine model and unable to persist *in vivo* (Torres et al., 2012). Similar findings were observed in a chicken infection model. An aerobactin-defective mutant of *iutA* was constructed and showed significantly decreased pathogenicity compared with the wild-type strain, as evidenced by the low extent of colonization in selected organs or being outcompeted *in vivo* (Gao et al., 2015). Moreover, a case–control clinical study found that *iutA* was an independent risk factor associated with 30-day mortality in patients with KP BSI. Consistent with previous results, we further determined that *iutA* carriage by causative strains was an independent risk factor for 30-day mortality in patients with KP BSI, and a higher mortality rate was observed when it was accompanied by MDR. We did not further analyze the risk factors of mortality in patients with CRKP BSI because of the limited number of cases. Notably, a fatal outcome was observed in patients with *iutA*-positive CRKP BSI (81.3%, 13/16). In addition, *iutA* was first found to be located on *pColV* plasmids, and subsequent studies revealed that it could also be chromosomally encoded in many strains (Warner et al., 1981; Lawlor and Payne, 1984; Mcdougall and Neilands, 1984). The emergence and dissemination of such hypervirulence plasmids, such as the virulence plasmid pLVPK that resulted in a fatal outbreak in ST11 CRKP (Gu et al., 2018), is concerning. Taken together, the *iutA*-related virulence factor plays a significant role in the pathogenesis of KP BSI and is a related marker associated with poor prognosis, especially in pathogenic strains accompanied by MDR and carbapenem-resistant phenotypes.

This study has several limitations. The retrospective nature of the study and the small sample size are intrinsic study limitations. In addition, the amount of missing patient data may have resulted in bias. Second, data from a single center limited generalizability to other geographical areas or institutions. Finally, although all the virulence genes presented in the tested strains were evaluated in our study, the expression of related genes was not determined in *in vitro* and *in vivo* experiments, such as knockout, qRT-PCR, and infection animal models.

In summary, this study identified that *iutA* or *Kvar_1549* gene was associated with poor prognosis, especially in MDR phenotypes of KP BSI. Moreover, the long-term colonization and dissemination of ST11-K64 carbapenem-resistant HvKP isolates resulted in increased difficulties and challenges in the treatment of infections. Therefore, clinicians should carefully manage patients with BSI caused by these difficult-to-treat strains. Further studies are needed to elucidate the pathogenic mechanisms and transmission dynamics of HvKP and carbapenem-resistant HvKP.

## Data Availability Statement

The original contributions presented in the study are publicly available. This data can be found here: the National Microbiology Data Center (https://nmdc.cn/en) under the accession numbers NMDC60014864-NMDC60014942.

## Ethics Statement

The studies involving human participants were reviewed and approved by the Ethics Committee of Lihuili Hospital, Ningbo Medical Center (no. KY2019PJ028). Written informed consent for participation was not required for this study in accordance with the national legislation and the institutional requirements.

## Author Contributions

XW, CH, and HW conceptualized and planned the work that led to the manuscript. QS and SS collected the clinical and MIC data. CH and XW analyzed the data. XW and CH drafted the manuscript. All authors contributed to the article and approved the submitted version.

## Funding

This work was supported by the Natural Science Foundation of Ningbo (Grant number 2019A610232).

## Conflict of Interest

The authors declare that the research was conducted in the absence of any commercial or financial relationships that could be construed as a potential conflict of interest.

## Publisher’s Note

All claims expressed in this article are solely those of the authors and do not necessarily represent those of their affiliated organizations, or those of the publisher, the editors and the reviewers. Any product that may be evaluated in this article, or claim that may be made by its manufacturer, is not guaranteed or endorsed by the publisher.
